# Fossil microbial shark tooth decay documents in situ metabolism of enameloid proteins as nutrition source in deep water environments

**DOI:** 10.1038/s41598-020-77964-5

**Published:** 2020-12-01

**Authors:** Iris Feichtinger, Alexander Lukeneder, Dan Topa, Jürgen Kriwet, Eugen Libowitzky, Frances Westall

**Affiliations:** 1 Geological-Palaeontological Department, Natural History Museum, Burgring 7, 1010 Vienna, Austria; 2 Central Research Laboratories, Natural History Museum, Burgring 7, 1010 Vienna, Austria; 3 Department of Palaeontology, University of Vienna, Geozentrum, Althanstraße 14, 1090 Vienna, Austria; 4 Department of Mineralogy and Crystallography, University of Vienna, Geozentrum, Althanstraße 14, 1090 Vienna, Austria; 5 CNRS, Centre de Biophysique Moléculaire UPR 4301, Rue Charles Sadron, CS 80054, 45071 Orléans, France

**Keywords:** Palaeontology, Bacteria, Palaeoecology

## Abstract

Alteration of organic remains during the transition from the bio- to lithosphere is affected strongly by biotic processes of microbes influencing the potential of dead matter to become fossilized or vanish ultimately. If fossilized, bones, cartilage, and tooth dentine often display traces of bioerosion caused by destructive microbes. The causal agents, however, usually remain ambiguous. Here we present a new type of tissue alteration in fossil deep-sea shark teeth with in situ preservation of the responsible organisms embedded in a delicate filmy substance identified as extrapolymeric matter. The invading microorganisms are arranged in nest- or chain-like patterns between fluorapatite bundles of the superficial enameloid. Chemical analysis of the bacteriomorph structures indicates replacement by a phyllosilicate, which enabled in situ preservation. Our results imply that bacteria invaded the hypermineralized tissue for harvesting intra-crystalline bound organic matter, which provided nutrient supply in a nutrient depleted deep-marine environment they inhabited. We document here for the first time in situ bacteria preservation in tooth enameloid, one of the hardest mineralized tissues developed by animals. This unambiguously verifies that microbes also colonize highly mineralized dental capping tissues with only minor organic content when nutrients are scarce as in deep-marine environments.

## Introduction

Teeth and bones are often the only evidence of ancient vertebrate life because of the mineralized nature of tissues. There are numerous possibilities for chemical alteration during the transition from the bio- to the lithosphere of which bacterial catabolysis of these tissues and organic matter within the carcass is an important example^1,2^. Preservation of soft tissue body fossils (e.g. skin) requires specific conditions of abiotic (e.g. salinity, temperature, pH-value, oxygen) and biotic (e.g. bacteria or bioturbation in general) factors, as well as marginal fluctuations in the continuum of processes during sedimentation, fossilization, lithification and preservation through geological time^3–5^. Paradoxically, microbial activity can be both preserving and destructive. One of the most famous preserving effects of microbial activity is documented by fossils from the Eocene Messel oil shale, which exhibit a unique preservation of soft tissues. Closer examination of the "skin shadows" preserved in these fossils reveals an accumulation of lithified bacterial colonies^3^, mirroring the original contour. However, the preservative effects of bacteria are, definitely, rare phenomena requiring specific conditions. Generally, microorganisms are predominantly responsible for destructive processes, removing digestible soft tissues of carcasses preceding diagenesis. Additionally, some organisms, like the bone-eating worm *Osedax*, literally invade bones to obtain nutrients^6^ when food supply is limited, as in bathyal marine settings.

Decay of bony material in modern deep-sea environments is dominated by anaerobic microbial decomposition of the large lipid reservoirs within bones^7^. Studies of decay processes in both modern and fossil deep-sea environments such as, whale-falls, show that they represent important nutrition supplies for deep-sea organisms. However, these previous studies focused only rarely on bacteria or archaea, which are at the base of the food webs^8^. Nevertheless, limitations in nutrients are also to be expected in ancient deep marine environments with similar microbial alteration of skeletal material, as in comparable modern habitats. Although microbial alteration of bones (bioerosion) is known from aquatic environments, equivalent alteration in teeth has only rarely been reported. Documented bioerosion patterns of dental tissues include endolithic macro- and microborings of fossil teeth and small tubules within dentine represented by traces and holes of endolithic bivalves, clionaid sponges, serpulid worms, and routes of microbial intrusion^9,10^. Here we review the fossil record of bioerosion and document for the first time in situ bacteria within the highly mineralized and organic-poor tooth enameloid of an extinct deep-water shark. This finding represents a hitherto unrecognized bioerosion type for teeth and nutrition source in deep-sea environments.

## Results

### Morphology

**Figure 1:**
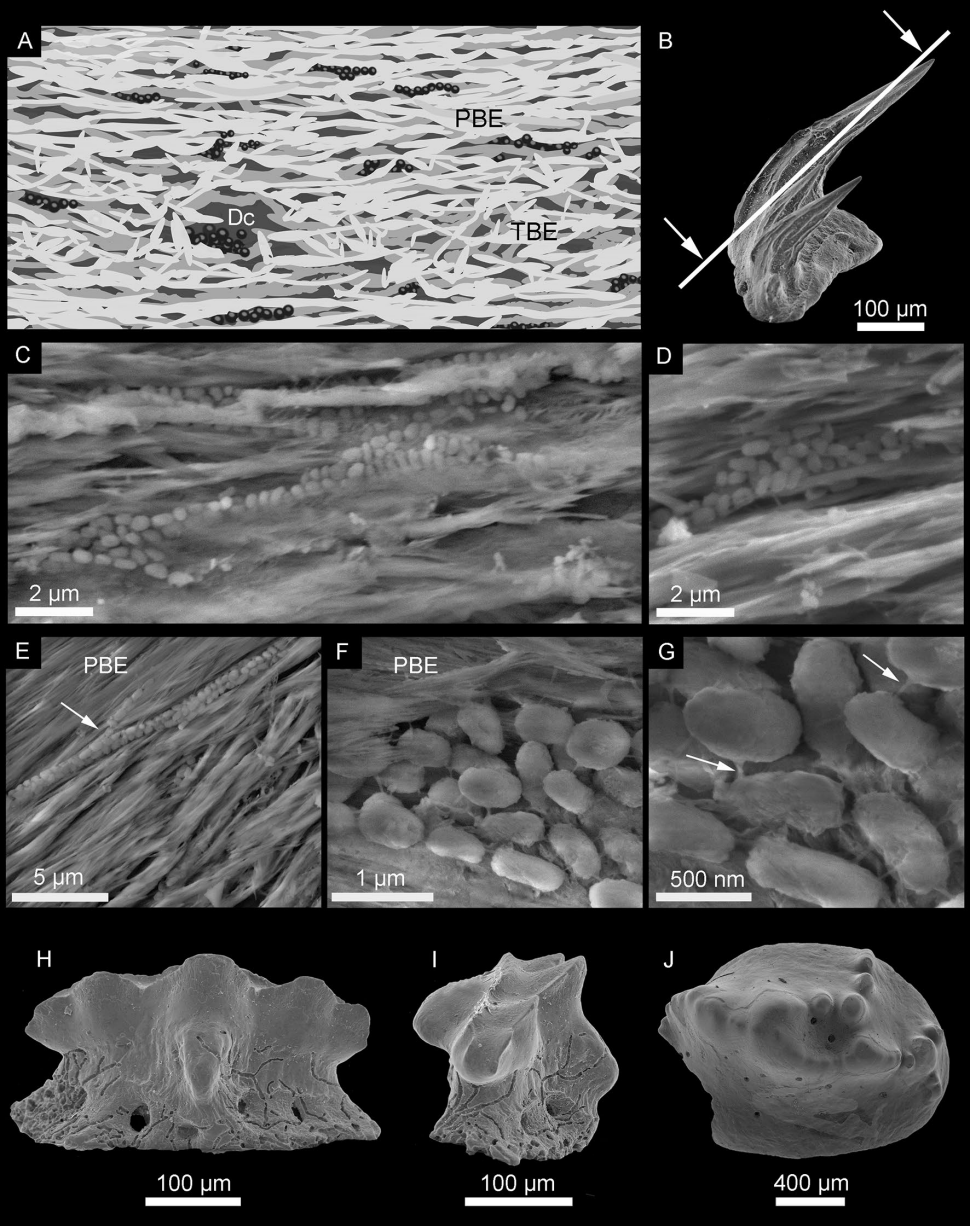
Sketch of bacteriomorph structures within the enameloid and scanning electron micrographs of fossilized bacteria of tooth NHMW 2017/0055/0028. (**A**) sketch of bacteriomorph bodies providing an overview about the frequency and arrangement of the coccoids within the enameloid bundles (for a better visualization, coccoids and enameloid bundles are not to scale). (**B**) holotype of *Cretacladoides noricum* (NHMW 2017/0055/0001) in profile view for demonstration of the section plane. (**C**–**E**) coccoid/rod-shaped bacteria arranged in chains embedded parallel to crystallite bundles of tooth enameloid. (**F**) denser associations in nest-like structures of fossilized bacteria. (**G**) close up of nest-like structures, white arrows indicate filmy substance. (**H**–**J**) Different teeth with examples of bioerosion on the tooth surface of the same sample. (**H**–**I**) Paratype of *Similiteroscyllium iniquus* (NHMW 2017/0058/0005) with branching type of bioerosion. (**J**) Pycnodont tooth with boreholes. Dc, Dentinal canal; PBE, Parallel Bundled Enameloid; TBE, Tangle Bundled Enameloid.

Based on the extensive enameloid investigations of previous studies^11^, several teeth of the extinct shark *Cretacladoides noricum* were studied in detail, of which only two out of 40 examined teeth display internal in situ microbial alteration of the superficial enameloid (Fig. 1C–G). Traces of bioerosion on tooth surfaces of various species of the same fauna are common despite the scarce evidence of internal alteration (Fig. 1H–J).

The enameloid of the teeth analyzed here is characterized by parallel to subparallel bundles of fluorapatite (Ca_5_(PO_4_)_3_F) and corresponds exactly to the pattern present in extant shark teeth^12^. Two of the teeth exhibit aggregates of mineralized, regularly shaped, coccoidal to short rod-shaped structures ranging from 0.5 to 1 µm in length and 0.4–0.5 µm in diameter. They are organized in chains that are arranged parallel to each other between the enameloid bundles (Fig. 1C,E). The surfaces of the coccoids are slightly irregular and most appear to be attached to each other and to the enameloid substrate by a delicate, filmy substance with a partially flaky appearance (Fig. 1F–G). Delicate fibrils can still be observed between the coccoidal structures (arrows in Fig. 1G). Some denser associations of aggregated structures also occur in spaces between the highly mineralized enameloid bundles (Fig. 1D,F) and within dentinal canals (Fig. 1A).

### Chemical composition

The coccoids and rods have a clearly mineralized appearance and are associated with two types of minerals: a compact mineral with a finely pitted surface that forms the body of the coccoids and rods, and tiny flaky minerals with a phyllosilicate appearance that are attached to the surfaces of many of the coccoids/rods and also to the filmy material that forms their immediate substrate (Fig. 1F–G).

**Figure 2:**
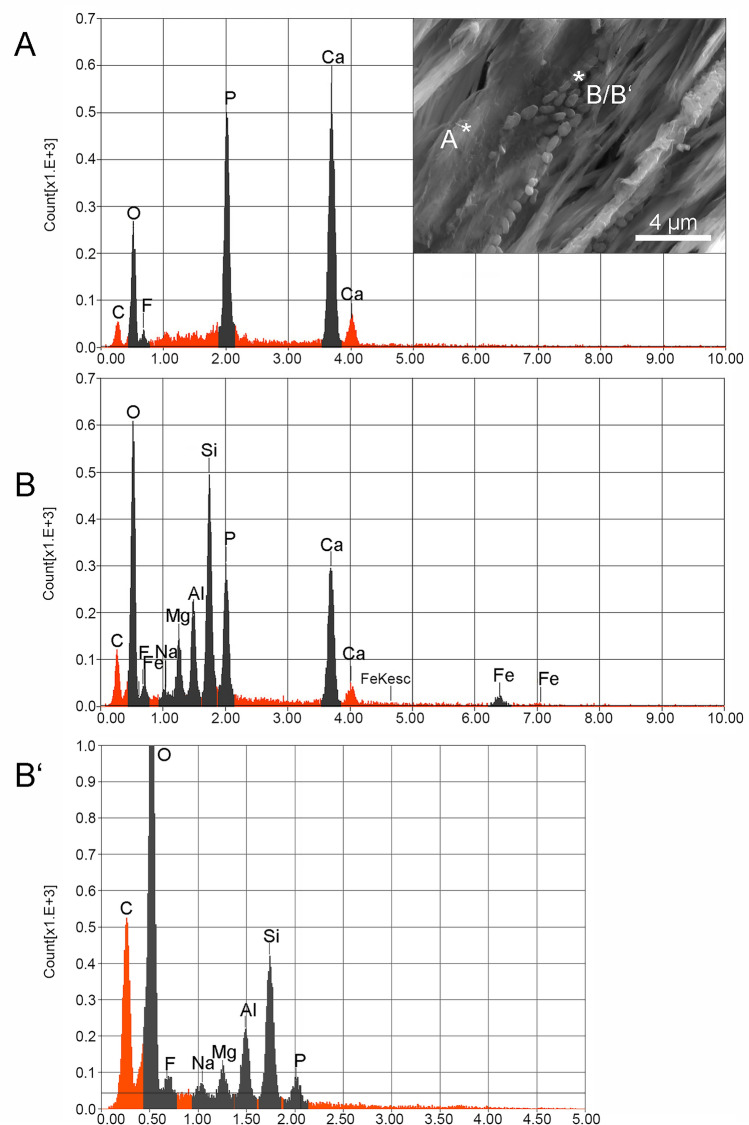
Quantitative energy dispersive spectrometry (EDS) of tooth NHMW 2017/0055/0028. (**A**) EDS analysis of fluorapatite crystal of tooth enameloid with 15 keV. (**B**) EDS analysis of fossilized bacteria with 15 keV. (**B**′) EDS analysis of fossilized bacteria with 5 keV.

EDS analysis of the highly mineralized enameloid bundle of one of the teeth at 15 keV at the NHM Vienna (Fig. 2A) shows distinct peaks of C (carbon coating), O, F, P, Ca (from left to right) resulting in stoichiometric oxide values of CaO (54.29 wt%), P_2_O_5_ (40.75 wt%), and F (4.96 wt%). After conversion to mol% and correction of oxygen valence by one fluorine, an apatite formula Ca_5.03_P_2.98_O_11.98_F_1.35_ close to the ideal composition is obtained. The chemical analysis of the coccoidal aggregates (Fig. 2B) indicates significant amounts of SiO_2_ (26.46 wt%), Al_2_O_3_ (9.57 wt%), FeO (5.45 wt%), MgO (5.01 wt%), and Na_2_O (0.75 wt%), in addition to CaO (27.56 wt%), P_2_O_5_ (23.60 wt%), and F (1.60 wt%).

The EDS analysis made at 15 keV clearly penetrated into the matrix and shows a compositional mixture of ~ 50% matrix and ~ 50% coccoidal structures, given their small size compared with the volume of excitation (by electrons) of the X-ray radiation (Fig. 2B). Using the Anderson-Hasler formula of X-ray range^13^, approximately 2 µm penetration depth must be considered at an average X-ray energy of 2 keV, a density of ~ 3 g/cm^3^ and 15 keV acceleration voltage. Thus, the results confirm the fluorapatite composition of the matrix but the Na, Mg, Al, Si and Fe peaks also indicate the presence of an aluminosilicate mineral (Fig. 2B). The composition presented in Table 1 (Spectrum B) therefore represents the combination of the chemistry of the matrix (= enameloid bundle) and the coccoidal aggregates (Fig. 2B). The analyses made at 5 keV, on the other hand, did not penetrate as deeply into the matrix (~ 0.25 µm using the Anderson-Hasler formula from above) and confirmed the presence of an aluminosilicate containing Na, Mg and Fe (L peak) (Fig. 2B′). These analyses corroborate the presence of a coating of phyllosilicate, probably a clay mineral close to smectite (montmorillonite) or saponite, encasing the bacteriomorph objects. Using the above composition without Ca and P (from the matrix), the formula (based on 4 Si) (Al_1.72_Mg_1.11_Fe_0.68_)[Si_4_O_10_] ∙ Na_0.25_ is obtained, which compares well with a clay mineral between montmorillonite, ~ (Al_1.67_Mg_0.33_)[(OH)_2_|Si_4_O_10_] ∙ Na_0.33_(H_2_O)_4_, and saponite, Mg_3_[(OH)_2_|(Si,Al)_4_O_10_] ∙ (Ca,Na)_x_(H_2_O)_y_^14^. Note that the 5 keV EDS analysis (Fig. 2B′) documents large C and O peaks, which are caused by a higher emission yield of light vs. heavier elements at low-energy (5 keV) excitation conditions. However, to test for the possible presence of reduced carbon entrapped within the mineralized structures of the coccoids, a comparative EDS study of enameloid and coccoids at 15, 10, and 5 keV was performed (see supplementary information for spectra).

**Table 1:** Results of chemical composition analyzed by EDS of a fluorapatite bundle of the tooth enameloid (Spectrum A) and a fossilized bacteria (Spectrum B) of the tooth NHMW 2017/0055/0028 with 15 keV and fossilized bacteria (Spectrum B′) with 5 keV.

Formula	mass%	mol%	Cation
**Spectrum A 15 keV**			
F	4.96	17.21	0
P_2_O_5_	40.75	18.94	0.96
CaO	54.29	63.85	1.61
Total	100	100	
**Spectrum B 15 keV**			
F	1.6	5.65	0
Na_2_O	0.74	0.8	0.04
MgO	5.01	8.36	0.18
Al_2_O_3_	9.57	6.31	0.28
SiO_2_	26.46	29.59	0.65
P_2_O_5_	23.6	11.17	0.49
CaO	27.56	33.02	0.73
FeO	5.45	5.1	0.11
Total	100	100	
**Spectrum B**′ **5 keV**			
F	4.16	13.85	0
Na_2_O	1.37	1.4	0.35
MgO	5.63	8.84	1.09
Al_2_O_3_	17.74	11.01	2.71
SiO_2_	54.69	57.59	7.09
P_2_O_5_	16.4	7.31	1.8
Total	100	100	

At 15 keV conditions, analysing for C, O, F, P, Ca independently (oxygen not by stoichiometry), the enameloid composition resulted in a formula of ~ Ca_5.07_P_3.00_O_12.13_F_0.81_ which is close to the ideal formula of apatite (see above). Carbon concentrations in enameloid were between 10.08 and 11.92 wt% in three spots. For comparison, two spots on the bacterial remnants resulted in 11.16 and 11.51 wt%. At 10 keV carbon contents on enameloid were scattered strongly between 6.54 and 16.90 wt%, whereas the coccoids gave 9.41 and 10.58 wt%. At 5 keV the carbon signal was strongly corrupted by the strong EDS zero peak and gave 31.47 wt% C in enameloid and 15.12 and 19.40 wt% in the bacterial structures. Thus, none of these measurements confirmed an excess of carbon from relics of organic material in the coccoids.

Contamination with recent bacteria can be excluded here because of the chemical fingerprint of extant bacteria, in which the three elements C, N, and O constitute 80–90 wt%^15^.

## Discussion

### Interpretation of the aggregates of coccoid/rod-shaped structures

The regular size and morphology of the coccoidal/rod-shaped structures in our study, as well as their specific distribution in linear chains in the cavities between the parallel bundles of tooth enameloid, is suggestive of microorganisms, such as bacteria. Indeed, they strongly resemble fossilized microorganisms associated with decaying macro-organisms elsewhere, such as the phosphatised bacteria of the “skin shadow” of fossil vertebrates in the Enspel oilshale^3,16^. Both extant and fossilized bacteria exhibit a size range between 0.5 and 4 µm^15–17^, which coincides with the size of the mineralized bodies interpreted as fossilized bacteria herein. Bacteria and cyanobacteria are the predominant bioerosion-causing organisms, however, both causal agents differ significantly in the type of traces they produce. Cyanobacteria are known to infest shells of marine bivalves in suitable habitats within the photic zone^18^. These phototrophic organisms create branching tunnels but do not produce traces with localized demineralization or cuffing of redeposited mineral, which is typical for terrestrial or freshwater bacteria^17,19,20^. Thus, the taphonomic alteration of the bacteriomorph invaders of the herein described shark teeth differs significantly from bacteria occupying terrestrial or freshwater environments. Nevertheless, the distinct morphology and size of the fossilized bodies point towards bacteriomorph microorganism but exclude cyanobacteria due to their phototrophic lifestyle.

Given the presence of bacteria, the observed filmy substance linking the individual bacteriomorph structures together, as well as the enameloid crystallites, are thus interpreted here as microbial extrapolymeric substances (EPS). EPS is a common exudate of microbes, used for attachment to substrates and as a control of the external physico-chemical conditions^21,22^.

As noted above, microbial degradation of organic substances is very common; what is not so common, however, is the physical preservation and mineralization of the degrading heterotrophs for which specific physico-chemical conditions are necessary, specifically, a micro-scale anaerobic environment (easily achieved through microbial oxidation of an organic substrate). Indeed, only two out of 40 tooth specimens exhibit this phenomenon in our study. The small number of affected teeth, however, indicates either very specific conditions under which invasion of bacteria into the hypermineralized tooth enameloid was feasible or lack of potential for fossilization of colonising microbes on or in other teeth due to diverging time intervals of tooth shedding, pH conditions, or other influencing factors.

It is not possible to establish the exact composition of the fossilized bacteria owing to their small size. Nevertheless, the morphology of the tiny flaky minerals attached to the surfaces of many of the coccoids/rods forming their immediate substrate is reminiscent of phyllosilicates replacing or formed on EPS-like film, as is also suggested by the elements typical for aluminosilicates in spectrum B (15 keV) and B′ (5 keV) of Fig. 2. In addition to Si and Al, the spectrum also documents the presence of Fe, Mg, and Na. If related to a phyllosilicate, this would indicate clay minerals close to smectite or saponite (see above). Substracting the clay composition from the spectra just leaves the coccoids/rods with compositions close to the fluorapatite matrix, although this may simply be a consequence of the excitation energy of the electron beam (15 and 5 keV) resulting in penetration through the very small fossilized structures into the background fluorapatite of the enameloid. Keeping in mind the fact that the samples were coated with carbon before SEM observation and EDS analysis, the carbon signal in the EDS spectra is easily explained. Beyond that, it is unlikely that the microorganisms were replaced by a carbonate because the C peak is too low.

Whatever the exact composition of the replacement minerals is, they are likely to have formed as the result of microbially influenced changes of the immediate environment leading to enrichment in certain elements with consequent precipitation onto functional groups of the degrading microbial structures^23^. During decomposition and diagenesis, the composition and concentrations of elements in the surrounding fluids control the type and composition of minerals replacing organic substrates. These elements come either from seawater and/or from elements released by the degradation of an organo-mineral substrate (mostly transition metals). Release of cell/EPS-bound elements is also influenced by the metabolic activity of microbes. For example, an increase in local alkalinity due to heterotroph degradation (by sulphate reducers) of primary photosynthetic mats releases Ca^2+^ ions into the fluid medium, which then combine with CO_2_ in seawater to form Ca carbonate^24^. In the case of the shark teeth in a deep-water environment, the organic substrate would be provided by the organic matrix (collagen and other proteins) of the teeth itself. If the replacing mineral was carbonate, the latter would have been enriched in transition elements, such as Fe and Mn (the latter not present at any detectable levels here), or Mg, resulting from the degraded substrate. In the case of phyllosilicate formation, Na-Fe^3+^ phyllosilicates have been experimentally produced on microbial EPS^25^. Indeed, EPS plays an important role in the biosynthesis of different types of clays^26^. Reactive sites on the surfaces of microbial cells act as loci for the nucleation of clay minerals in the poorly crystalline state^27^. Subsequent diagenesis and aging transforms these poorly crystalline materials into crystalline phases.

### Enameloid invading bacteria

The presence of bacteria associated with teeth is, during the lifetime of an animal, normal but they also contribute to microbial degradation of soft-tissues after death during decomposition^3–5^. Normally, bacteria are only found on surfaces or within the pulp cavity, which is easily accessible. However, they also gain access to tissues such as dentine through tooth surface lesions or due to previous bioerosion of other organisms. It is thus not surprising to find their fossilized remains associated with skeletal structures such as shark teeth and even within dental tissues. However, the presence of bacteria within the hypermineralized capping tissues of teeth, such as enamel or enameloid providing only very small amounts of severe accessible organic matter as a possible nutrition source, has not been documented up to now.

The resistant hypermineralized outermost layer of enameloid of shark teeth consists of fluorapatite. The fluorapatite crystals are embedded in an organic matrix of about 4.5 wt%, as documented for a tooth of a great white shark (*C. carcharias*)^28^ indicating a variable content of collagen, proteins, and other organic structures (e.g. tubular vesicles) in enameloid depending on species and tooth maturation^29^. It has been demonstrated that the fluorapatite crystallites are devoid of any organic matter, while the crystallite bundles are encased by an organic matrix that generally has a smooth, sometimes also a fibrous appearance^12^. Additionally, the collagen fibres within the enameloid of an extant salmon shark (*L. ditropis*) are arranged in a regular pattern and the fibres cross each other^30^.

Collagen plays a special role in the composition of all structures needed for many eukaryotes, from plesiomorphic sponges to vertebrates^31^, and is significantly resistant to post-mortem decay^19^. Two types of collagen, un-mineralized and mineralized, whose resistance to deterioration are vastly different^20,32^, occur in different maturation states of teeth and bones. Apart from the rapidly degradable un-mineralized collagen type of primarily fresh bone, specific conditions are required for the assimilation of mineralized collagen, which is the type found in hypermineralized tooth capping tissue and mature bone. Here, the isolated collagen fibres are stabilized by tiny hydroxylapatite platelets responsible for avoiding direct enzymatic degradation. Thus, removal of the densely packed mineral platelets is necessary for effective microbial cracking of the large collagen molecules^19,32,33^.

A comparative process of tooth decay is caused by the demineralization causing caries in human teeth. Caries-causing bacteria that occur in plaque ferment carbohydrates (e.g. glucose and fructose) and generate an acidic microenvironment, which has the ability to demineralise the enamel^34^. Only as a result of this process, bacteria gain access to the now un-mineralized tissue for utilization the matrix proteins of enamel and dentine^34,35^. Thus, any kind of tooth decay is directly linked to low pH values or acidic microenvironments. As noted above, it is in such low pH environments that elements, such as Ca, can be released from an organic substrate and subsequently re-precipitated as a mineral^24^.

Accordingly, the organic matrix between the fluorapatite bundles inside the teeth would have provided a nutrient supply for invading microbes. In bones, microbial degradation of collagen fibrils using collagenases provides a high-energy yield^36–38^ and bacteria invade the bone through haversian canals in order to attain this valuable nutrient supply^39^. While they subsequently follow the collagen fibrils, they are unable to cross the cement lines of secondary osteons^19^. Similarly, microbes can invade shark teeth via the nutritive foramina in the root and ascend apically using dentinal canals, resulting in a chain-like arrangement of bacteria. Moreover, bacteria can migrate into the enameloid because the enameloid/dentine boundary is not an insuperable separating layer in chondrichthyans, as is the enamel/dentine boundary in mammals, but is penetrated by dentinal tubes extending into the enameloid. This would form possible pathways for bacteria from the dentine into the enameloid corresponding to the orientation, arrangement and location of the fossilized bacteria between the enameloid bundles.

However, invasion of enameloid by means of external bioerosion of the tooth surfaces, which is observable in numerous teeth of different species deriving from the same faunal assemblage (Fig. 1H–J), enables an easily accessible entrance directly through the external single crystallite layer of the enameloid. Considering all possible pathways of penetration, intrusion facilitated by surface lesions caused by bioerosion or even as symbiont of macro-organisms presents another plausible scenario.

Numerous incredible symbiotic relationships are known in the biosphere, resulting in a benefit of both involved parties. An obvious example of a remarkable symbiosis of a comparative, marine habitat represents the bone-eating worm *Osedax,* which hosts microbial symbionts to benefit from their collagenolytic enzyme activity^40^. The symbionts comprise primarily *Oceanospirillales* and *Epsilonproteobacteria*, which colonies the root tissues of the small worms enabling the degradation of different types of collagen during intrusion of the worm into bones of a whale fall^40^. Indirect evidence provided by trace fossils (i.e. boreholes) in fossil whale bones, document that this highly specialized polychaete worm has a fossil record since the Oligocene (~ 30 million years)^6^, but most likely already originated in the Cretaceous^41^.

Comparing the borehole diameter generated by recent *Osedax* species (e.g. *O. rubiplumus*) and ancient traces measured on fossil whale bones a possible trend in borehole size increasing from the Oligocene (0.1–0.45 mm) to today (0.1–2.0 mm) is recognized^6^. Thus, the slightly smaller (~ 0.08 mm) boreholes in teeth of this fauna (Fig. 1J) could be considered to result from an ancestral representative of *Osedax* or a similar organism, which distinctly possessed the ability to digest collagen and other proteins due to microbial symbionts^40^. Although this remains rather hypothetical, the ability to digest collagen using collagenolytic enzyme activity in the organic-poor, oligotrophic environment of the deep-sea, is of well-documented benefit to some microbes such as, e.g., *Oceanospirillales*^40^.

It is unclear, which organism is responsible for the bioerosion patterns (boreholes) present in the fossil teeth, such as a pycnodont fish tooth (Fig. 1J) from this locality, as well as the exact process of demineralization of the mineralized collagen that is necessary for digestion of the small amount of collagen. However, some recent microorganisms, such as *Streptococcus mutans*, which is responsible for tooth decay in humans^42^, and *Oceanospirillales* or *Epsilonproteobacteria* (common symbionts of the bone-eating *Osedax*), possess the ability to digest different types of collagen^40^. Despite the fact that these are only a few examples of microbial species, which are able to obtain nourishment from mineralized collagen, they show that some specialists adapted to this food source and also survived in extreme habitats.

Cyanobacteria also produce collagenases to tunnel into marine shells^18^, but their phototrophic lifestyle precludes them as candidate organisms for degradation of shark teeth in a deep-sea setting. Additionally, cyanobacteria normally form branching tunnels rather than creating chain-like arranged globular structures. Consequently, considering different modes of life and especially environmental limitations, we argue for a heterotroph lifestyle of the herein described bacteria.

### The fossil record of bioerosion

Bioerosion results in the loss of information and thus plays a crucial role affecting the structure and consequently the potential for preservation of hard tissues like bones and teeth. Nevertheless, microbial alteration of skeletal structures provides much evidence about taphonomic conditions. Bioerosion caused by microbes is a phenomenon that is known since at least the nineteenth century when the anatomist and histologist Rudolf Albert von Kölliker described meandering tunnels in e.g. a fossil gastropod (*Aporrhais pespelecani*) and in a Cretaceous fish scale (*Beryx ornatus*)^43^. However, subsequent studies of the Viennese Pathologist Carl Wedl, focusing on tunnel-like structures in human teeth, had received much more publicity^44^. Kölliker attributed these tunnels to a fungal attack, while Wedl is not specific about the causal agents and described them either as parasitic plants, microscopic parasites, or as fungi^43,44^. Further investigations by Wedl additionally demonstrated the occurrence of these microstructures in a horse bone, in teeth of fossil cartilaginous fishes (*Hemipristis* and *Myliobates*), and one bony fish (*Pycnodus*)^44^. However, the interpreted fungi do not penetrate the enamel, being limited to the spongy bone and tooth cementum^9,44,45^. Following investigations on bone (e.g. *Nothosaurus*, *Plesiosaurus*, and *Ichthyosaurus*) and cartilage (e.g. *Squatina*, *Galeocerdo*, and *Carcharias*) in various groups, Roux^46^ described the bone-penetrating fungus, *Mycelites ossifragus*. Subsequent investigations led Bernhauser^47^ to conclude that the structures described by Wedl^44^ and Roux^46^ belong to an inchnogenus rather than a distinct fungal species.

Hackett^48^ was the first to identify bacteria as causal agents for different structures described in teeth and bones, thus introducing a Wedl-type (apical expansion of meandering, bifurcating tunnels) and three non-Wedl-types (linear longitudinal, lamellate, and budded foci) of structure. Of these four types of bioerosion structures, only the Wedl-type is supposed to originate via fungal colonization and the other three types are interpreted to be the product of bacterial activities.

Since the fundamental contributions of Wedl^44^, Roux^46^, and Hackett^48^, numerous studies have dealt with histological microstructures of exhumed teeth and bones of ancient humans^39,45,49–51^, marine and terrestrial vertebrates^6,8^ and marine invertebrates^52,53^. The focus of some studies was on causal agents^46,48^, others, conversely, examined the influence of different environmental settings and the subsequent impact of the alteration of the tissue by specific micro-organisms^45,54,55^. However, the identity of the microorganisms responsible for bioerosion patterns in teeth and bones usually remains ambiguous^54^.

Fossil bacteria are well known throughout the rock record and are the oldest known preserved traces of life, the latter occurring as silicified remains^56–58^. In rare cases, fossilized in situ colonies are documented, e.g., in various Eocene vertebrate fossils with soft tissue preservation from the Messel pit^16^, in an Early Cretaceous pterosaur head crest from Brazil^59^, and in the dentine of a historic human tooth^45^. Another example of soft tissue preservation replicated by microbial biofilms is the conservation of muscle fibres in a Jurassic horseshoe crab^60^. However, in situ bacteria invading tooth enameloid, one of the hardest and most highly mineralized biogenic tissues developed by an animal and lacking significant cavities or lacunae in contrast to dentine, have not been reported up to now.

## Conclusion

We identified fossilized microorganisms inhabiting the hypermineralized outermost layer of enameloid of teeth in the extinct, Early Cretaceous shark, *Cretacladoides noricum*. The 0.5–1 µm-sized organisms, associated in linear, chain-like colonies occur in the enameloid of the shark teeth between the parallel bundles of the fluorapatite crystallites.

A delicate filmy substance coating and linking the fossilized bacteriomorph bodies was replaced by a Fe–Mg rich phyllosilicate, most probably a clay mineral close to smectite (montmorillonite) and saponite. However, limitations of the analytical conditions preclude definitive identification of the mineral phase replacing the microfossils.

The microorganisms were likely fossilized during decay and diagenesis of the shark teeth since microbial invasion of bones and teeth (reservoirs of proteins) is a known behaviour of heterotrophs in the oligotrophic deep-sea environment. This observation is particularly significant because it is the first time that fossilized microorganisms have been observed in the highly mineralized enameloid of shark teeth. Moreover, it shows that microbes obviously not only colonize less mineralized skeletal structures such as bones or dentine, but also target the scarce organic matter (collagen and other proteins) in highly mineralized tissues such as enameloid if the boundary is permeable or the surface is damaged. Even though the organic content in the enameloid is rather low, it seemingly nevertheless provides an additional, high-energy source of nutrient matter in an otherwise nutrient-poor environment.

## Materials and methods

### Material

The bacteria-bearing teeth forming the focus of this study belong to the extinct, Early Cretaceous shark species, *Cretacladoides noricum*^11^ and were found in association with the published Early Cretaceous deep-water chondrichthyan assemblage reported in Fuchs et al.^61^ and Feichtinger et al.^11,62^.

### Methods

The teeth were extracted from the limestone matrix using 12% acetic acid (W. Neuber`s Enkel, Vienna). The residual sediment was screen washed using different mesh sizes (500, 250, 125, and 63 µm) and dried by 60 °C for 24 h. The extraction process was made on two, separate samples in order to exclude contamination. The teeth were separated from the residual sediment using a fine preparation needle and were mounted in resin (Körapox 439, Kömmerling) in longitudinal direction, subsequently wet-ground with siliciumcarbid 1000 (~ 4 µm grain size) and the resulting surface (as indicated in Fig. 1B) polished with Micropolish II Alumina 0.3 µm Powder (Buehler, U.S.A.), and finally etched with 10% HCl (W. Neuber`s Enkel, Vienna) for five seconds. In a next step, the samples were rinsed for 2 min in distilled H_2_O, cleaned with an ultrasonic bath for 5 min, and dried by 60 degrees for 2 h. Tooth NHMW 2017/0055/0028 was then examined with a FEI Quanta 3D FEG at the Department of Lithospheric Research at the University of Vienna, without any coating with an excitation energy of the electron beam of 5 kV. Quantitative energy dispersive spectrometry (EDS) analysis of the enameloid bundle and aggregates of tooth NHMW 2017/0055/0028 were obtained by a JEOL “Hyperprobe” JXA 8530-F field-emission electron microprobe (FE-EPMA) in combination with an online JEOL quantitative ZAF-correction program at the Central Research Laboratories of the Natural History Museum Vienna (NHMW). For the EDS analyses, the sample was coated with a 10 nm carbon film. An accelerating voltage of 15 and 5 keV, a beam current of 5 nA, and fully focused electron beam (with an estimated beam diameter of ~ 70–80 nm) were used. The Count Rate was 1055.00 CPS. The comparative EDS studies of enameloid and coccoids were performed with a FEI Inspect-S scanning electron microscope with an EDAX Apollo XV SDD EDS detector at 15, 10 and 5 keV acceleration voltage. Spectra were acquired for 30–90 s to obtain a good signal to noise ratio, and intensities were corrected with the ZAF algorithm. The teeth (NHMW 2020/0042/0001 and NHMW 2017/0055/0028) are housed in the Geological—Palaeontological Department of the Natural History Museum Vienna, Austria (NHMW). The brightness and contrast of the images were adjusted using Adobe Photoshop Elements 8.0. Ink.

### Geological setting

**Figure 3:**
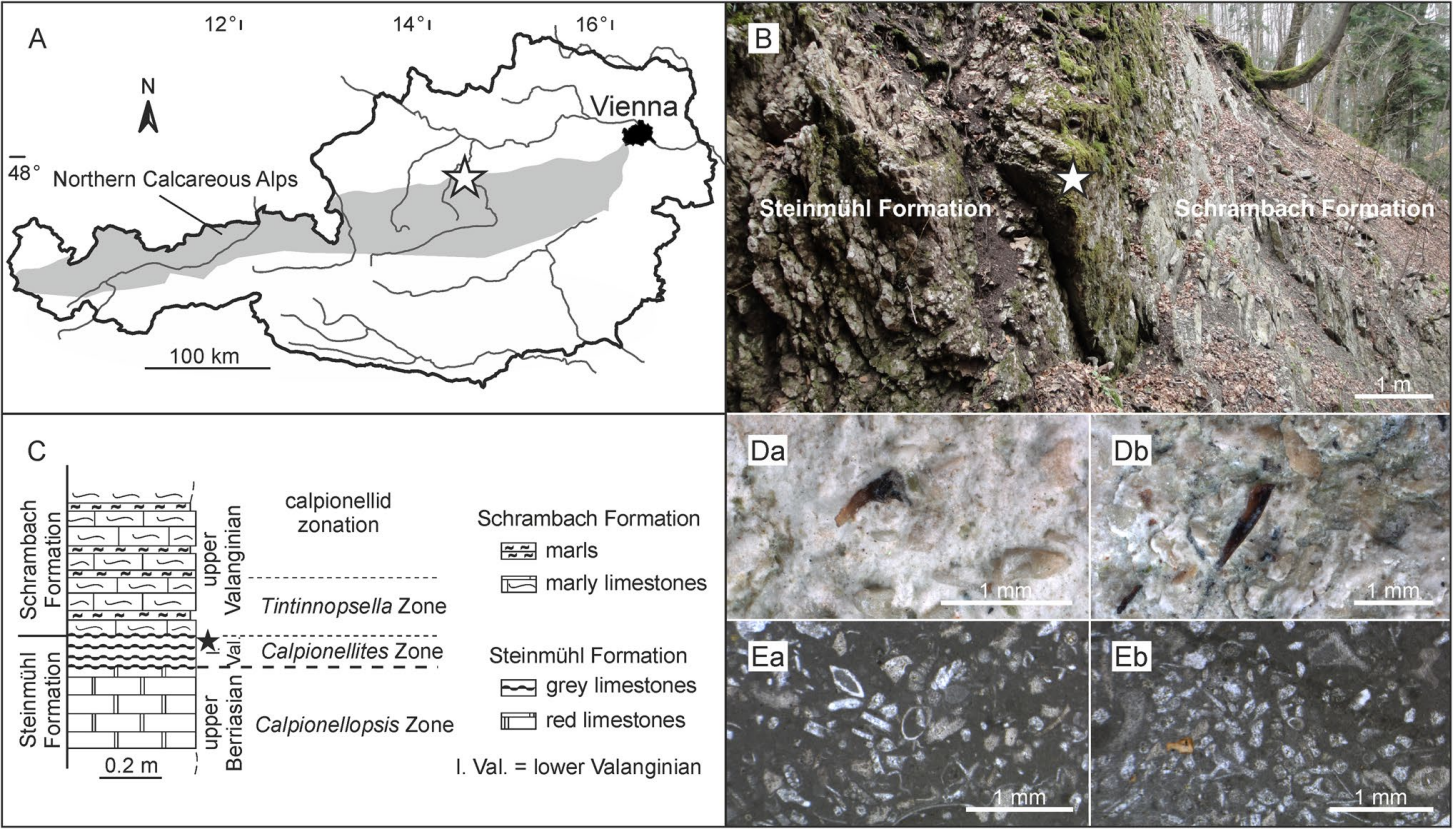
(**A**) locality map of the Klausrieglerbach 1 section (KB1-A) in the Northern Calcareous Alps of Upper Austria with the indicated fossil locality (white star). (**B**) KB1-A outcrop with the older red Steinmühl Formation (left) and the grey Schrambach Formation (right). (**C**) lithologic and stratigraphic column of the KB1-A section with indicated shark teeth layer (black star). (**Da**) and (**Db**) shark teeth on naturally dissolved rock surface. (**Ea**) and (**Eb**) thin sections of the shark teeth bearing bed. (**Ea**) bioclastic wackestone, mud supported, with crinoid fragments, ammonites, ostracods, bivalves, and foraminifera. (**Eb**) bioclastic wackestone to packstone, partly mud or grain supported, with crinoid fragments, ammonites, ostracods, bivalves, and foraminifera (note two fragments of shark scale or teeth in left lower area).

The teeth described here derived from the KB1-A section that consists of bioclastic wacke- to packstones of the so-called Steinmühl Formation occurring in the northern tectonic units of the Northern Calcareous Alps in Upper Austria (Fig. 3A–C). The exact position of the KB1-A section was determined by global positioning system (E 14°21′10″, N 47°54′32″) and is dated as upper Berriasian to lower Valanginian^63^. The teeth-yielding rock comprises abundant remains of crinoids, aptychi, bivalves, foraminifera, ostracods, radiolaria, and significant calpionellids (Fig. 3D,E). Ammonites, belemnites and brachiopods dominate the macrofossil content, with the extraordinarily frequent teeth of the extinct shark genera *Cretacladoides*, *Natarapax*, *Altusmirus*, *Fornicatus*, and *Similiteroscyllium* comprising five percent of the rock volume. Additionally deeper water bivalves and pelagic foraminifera (planktonic favusellids) hint to open marine conditions and deeper depositional environments for the condensed shark tooth layer. Condensation took place in deep marine areas by deep-water currents and winnowing of sediment, leading to condensation and enrichment of bioclastic material. The assumed deep-water environment is also mirrored by the presence of the microfossil group of calpionellids typical for pelagic to hemipelagic sedimentation. The facies and fossil assemblage from macro- and microfossils observed in thin sections is also characteristic for deep-water pelagic deposits and basinal settings from 200 to 1000 m in the Tethyan Lower Cretaceous^63,64^.

## Supplementary information

Supplementary Informations.
