# Supplementary material for: Fossil microbial shark tooth decay documents in situ metabolism of enameloid proteins as nutrition source in deep water environments
Source: Sci Rep. 2020 Dec 1;10:20979. doi: 10.1038/s41598-020-77964-5 (PMC7708646; doi:10.1038/s41598-020-77964-5)
Supplement: Supplementary file 1 — Supplementary Informations. [file 41598_2020_77964_MOESM1_ESM.pdf]

Supplementary information for:

Fossil microbial shark tooth decay documents *in situ* metabolism of enameloid proteins as nutrition source in deep water environments

Feichtinger I., Lukeneder A., Topa D., Kriwet J., Libowitzky E., and Westall F.

EDS spectra and analytical results of the comparative EDS study at 5, 10, and 15 keV of the bacterial remnants (pages 1 - 6) and the matrix enameloid (pages 7 - 13). The comparison of spectra at different excitation voltages confirms the relative attenuation of heavier elements and enhancement of light elements at lower voltages. At a certain voltage, the carbon contents of the bacterial remnants scatter indistinguishably among those of the enameloid matrix (15 and 10 keV), or are even below with strong scatter at 5 keV (note the interference with the zero-energy peak).

5 keV Bacteria Page 1

5 keV Bacteria Page 2

10 keV Bacteria Page 3

10 keV Bacteria Page 4

15 keV Bacteria Page 5

15 keV Bacteria Page 6

5 keV Enameloid Page 7

10 keV Enameloid Page 8

10 keV Enameloid Page 9

10 keV Enameloid Page 10

15 keV Enameloid Page 11

15 keV Enameloid Page 12

15 keV Enameloid Page 13

Supplementary information for:
Fossil microbial shark tooth decay documents in situ metabolism of enameloid proteins as nutrition source in deep water environments

Feichtinger I., Lukeneder A., Topa D., Kriwet J., Libowitzky E., and Westall F.

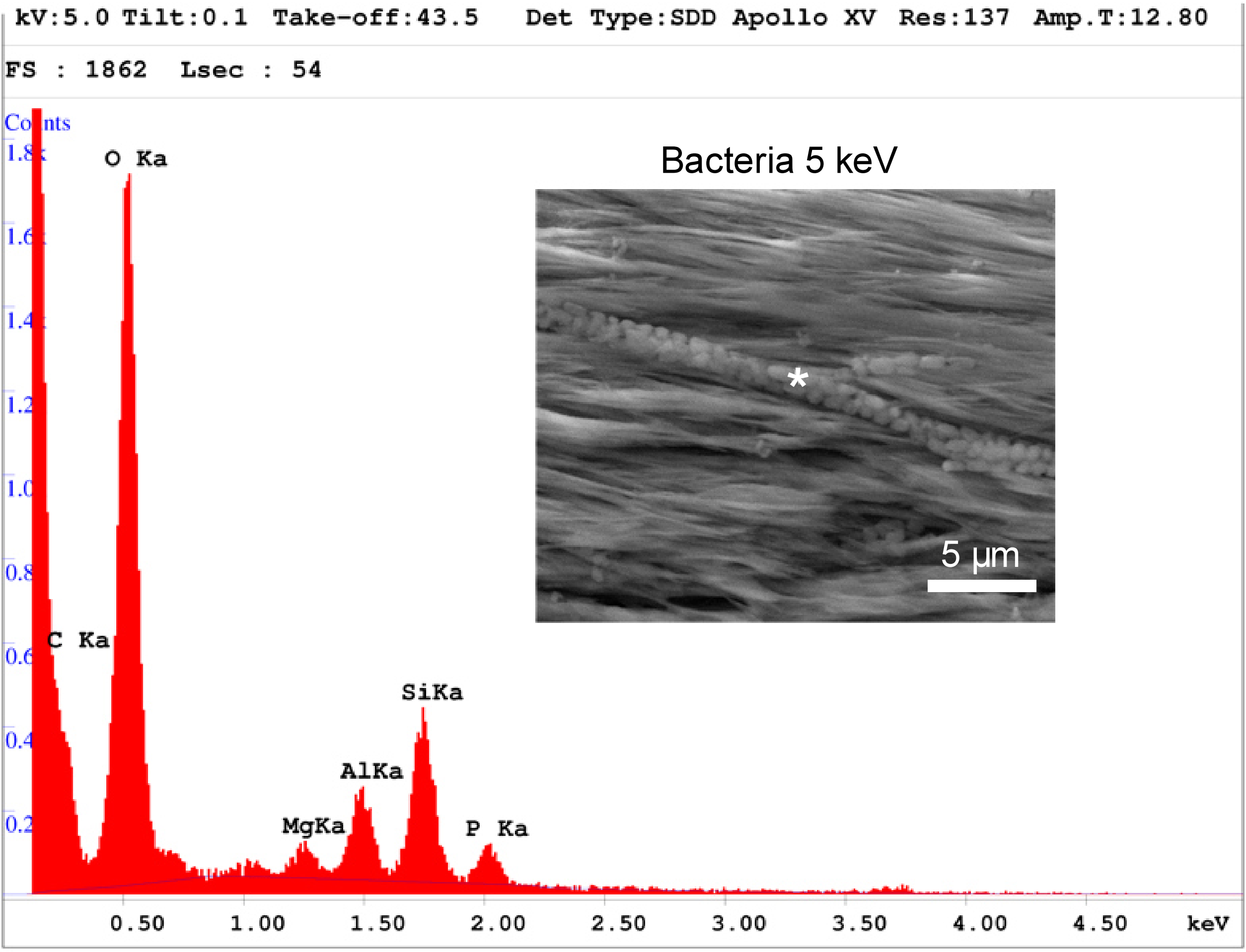

Element	Wt %	At %
C K	15.12	23.64
O K	38.66	45.38
MgK	2.80	2.16
AlK	10.39	7.23
SiK	25.03	16.74
P K	7.99	4.85
Total	100.00	100.00

Element	Net Inte.	Bkgd Inte.	Inte. Error	P/B
C K	46.30	1.53	2.05	30.31
O K	203.63	3.09	0.96	65.86
MgK	10.49	6.27	6.17	1.67
AlK	31.24	6.18	2.85	5.05
SiK	57.25	5.31	1.94	10.78
P K	13.26	4.38	4.77	3.02

c:\edax32\genesis\genspc.spc

Label:

kV:5.0 Tilt:0.1 Take-off:43.5 Det Type:SDD Apollo XV Res:137 Amp.T:12.80

FS : 1704 Lsec : 52

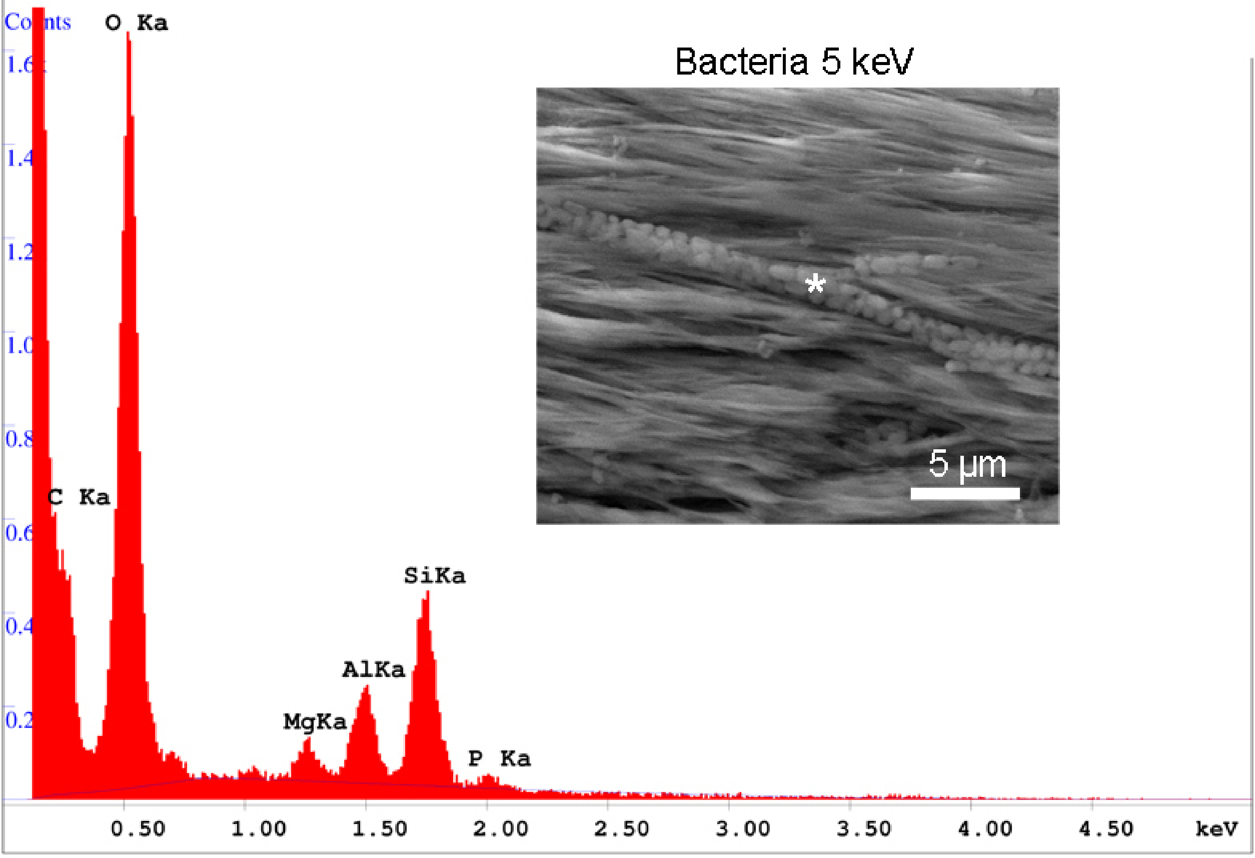

Element	Wt %	At %
C K	19.40	29.28
O K	37.44	42.42
MgK	3.02	2.25
AlK	10.55	7.09
SiK	27.35	17.65
P K	2.24	1.31
Total	100.00	100.00

Element	Net Inte.	Bkgd Inte.	Inte. Error	P/B
C K	63.42	1.83	1.78	34.71
O K	199.72	3.67	0.99	54.37
MgK	11.52	6.85	6.02	1.68
AlK	32.26	6.66	2.89	4.84
SiK	63.55	5.61	1.88	11.32
P K	3.77	4.51	13.09	0.84

c:\edax32\genesis\genspc.spc

Label:

kV:10.0 Tilt:0.0 Take-off:43.4 Det Type:SDD Apollo XV Res:137 Amp.T:12.80

FS : 2443 Lsec : 67

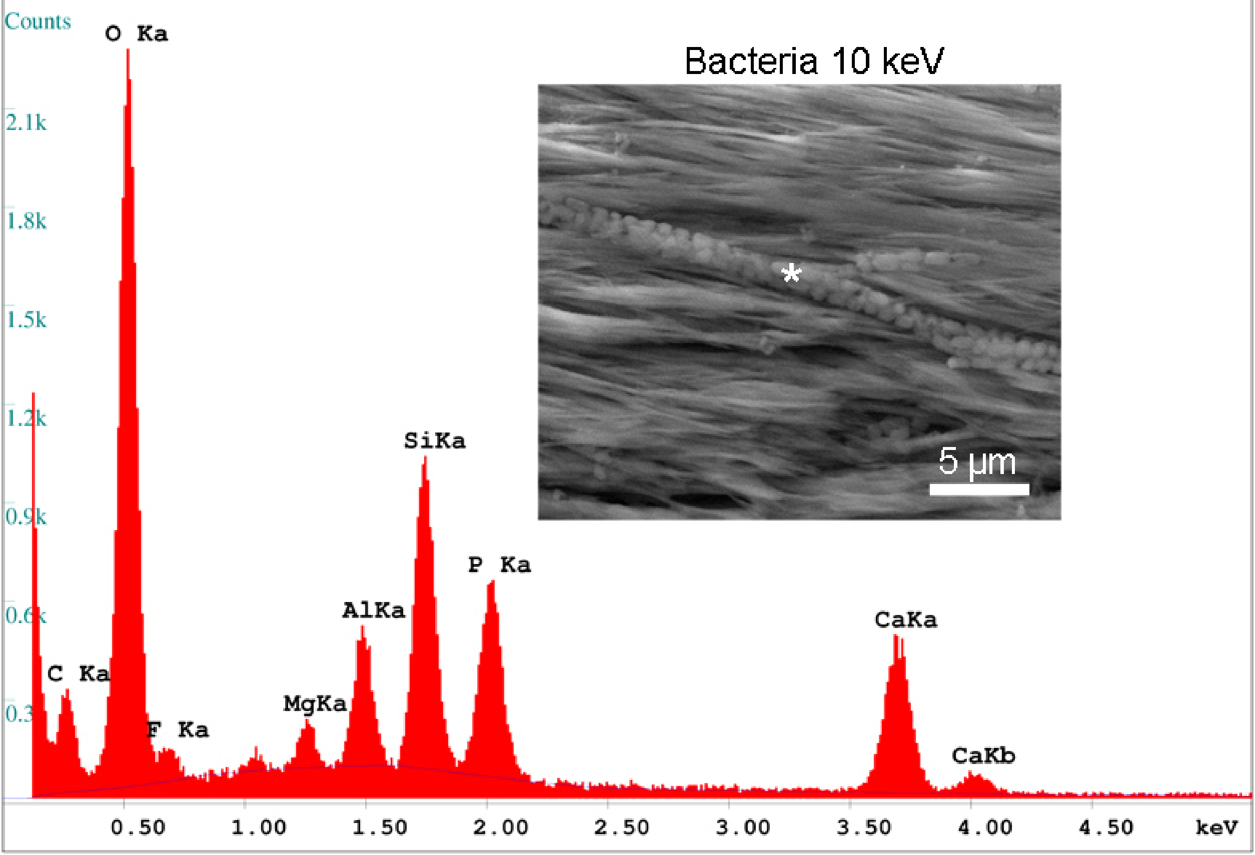

Element	Wt %	At %
C K	9.41	15.57
O K	48.19	59.88
F K	2.95	3.08
P K	12.97	8.33
CaK	26.48	13.14
Total	100.00	100.00

Element	Net Inte.	Bkgd Inte.	Inte. Error	P/B
C K	30.40	2.17	2.36	13.98
O K	216.52	4.20	0.84	51.55
F K	10.97	6.12	5.34	1.79
P K	73.22	9.67	1.60	7.57
CaK	67.98	2.87	1.54	23.69

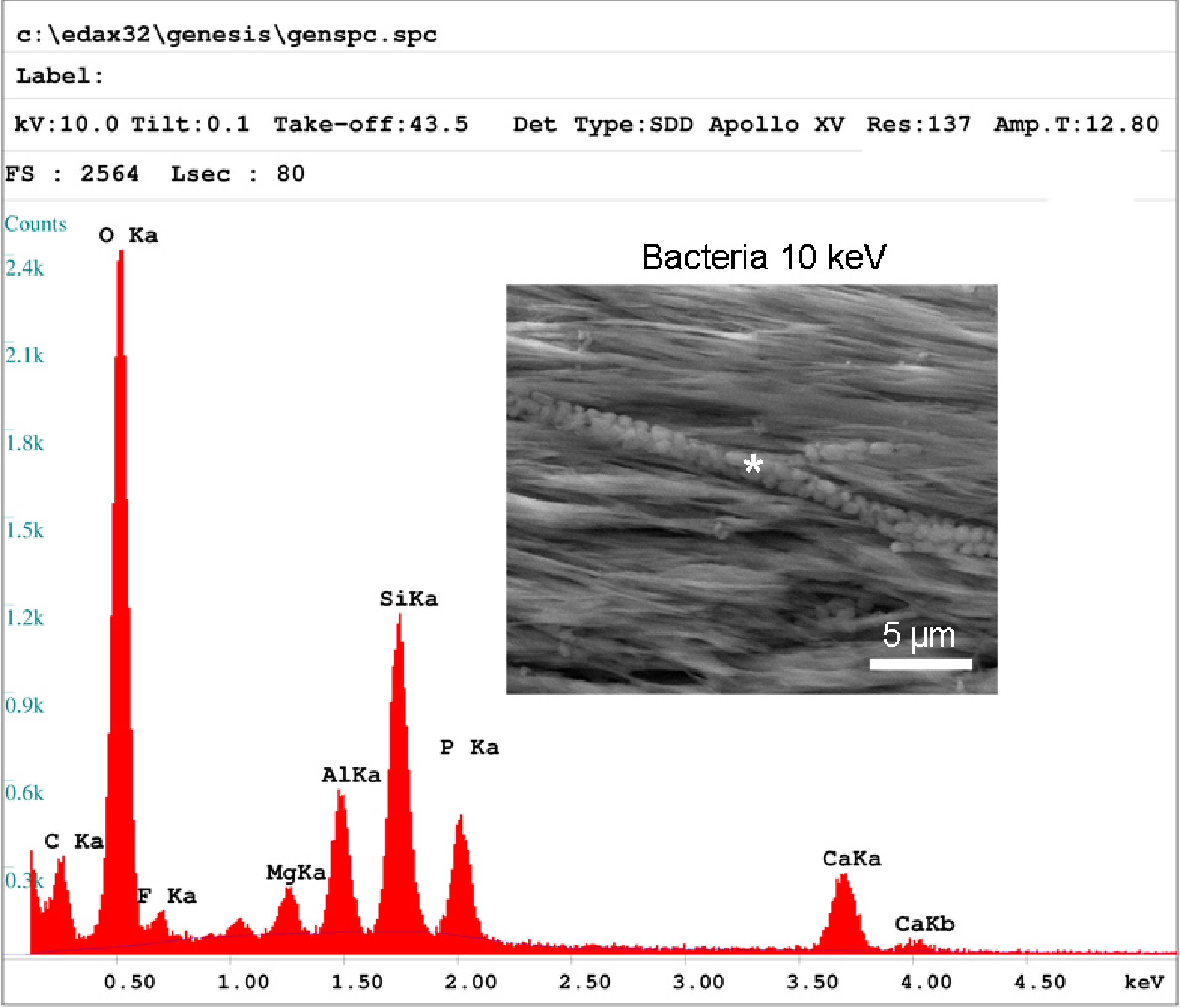

Element	Wt %	At %
C K	10.58	16.32
O K	56.28	65.14
F K	3.48	3.39
P K	10.66	6.38
CaK	19.00	8.78
Total	100.00	100.00

Element	Net Inte.	Bkgd Inte.	Inte. Error	P/B
C K	24.04	1.43	2.41	16.76
O K	192.84	2.79	0.82	69.01
F K	8.48	4.08	5.37	2.08
P K	39.33	7.92	2.11	4.97
CaK	31.93	2.16	2.11	14.80

c:\edax32\genesis\genspc.spc

Label:

kV:15.0 Tilt:0.1 Take-off:43.5 Det Type:SDD Apollo XV Res:137 Amp.T:12.80

FS : 1589 Lsec : 58 10-Sep-2020 13:22:34

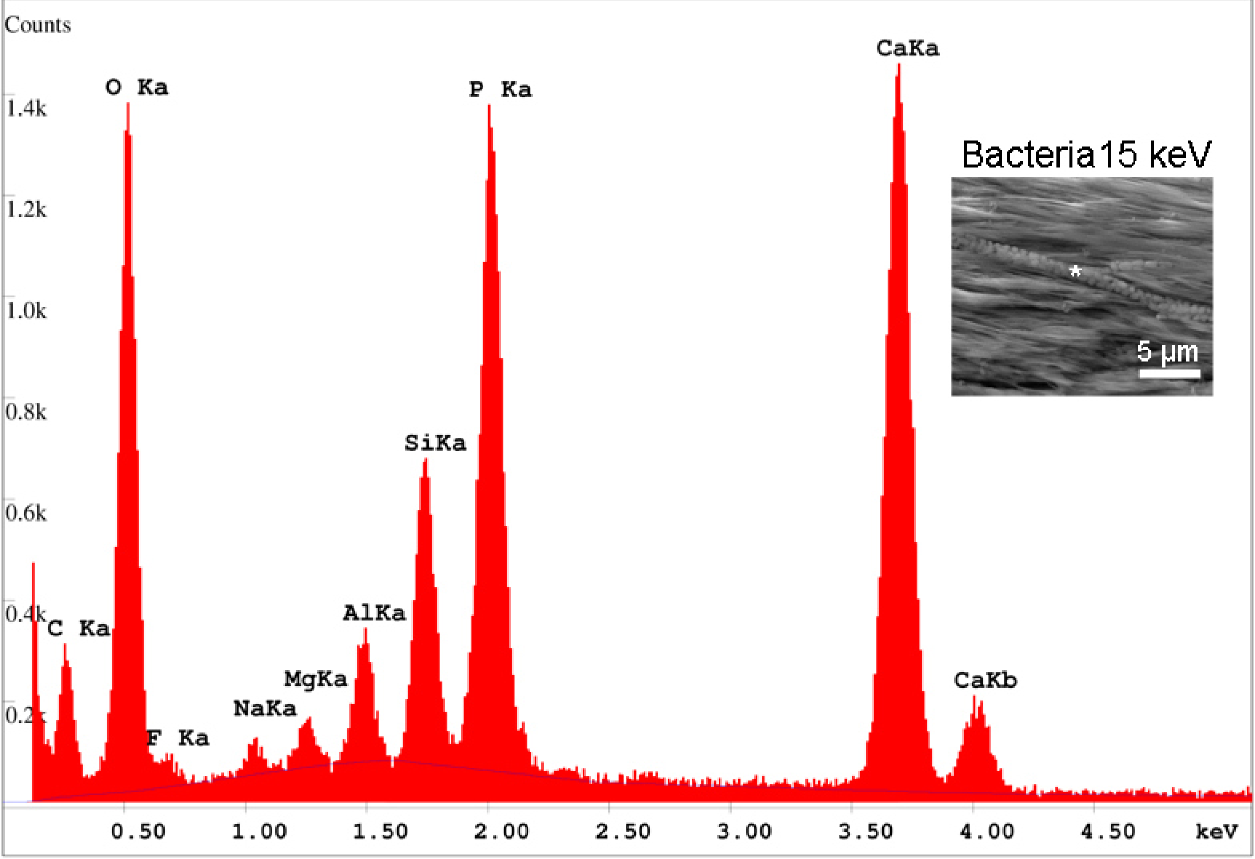

Element	Wt %	At %	Element	Net Inte.	Bkgd Inte.	Inte. Error	P/B
C K	11.51	20.31	C K	30.63	1.55	2.49	19.80
O K	34.24	45.37	O K	148.34	2.92	1.10	50.77
F K	1.61	1.80	F K	7.20	4.18	7.18	1.72
NaK	0.71	0.65	NaK	7.61	8.64	8.59	0.88
MgK	0.85	0.74	MgK	11.51	10.81	6.55	1.07
AlK	2.32	1.82	AlK	33.51	13.89	3.06	2.41
SiK	5.89	4.45	SiK	83.39	13.30	1.65	6.27
P K	14.01	9.59	P K	179.38	10.71	1.04	16.75
CaK	28.87	15.27	CaK	237.64	4.50	0.87	52.77
Total	100.00	100.00					

c:\edax32\genesis\genspc.spc

Label:

kV:15.0 Tilt:0.1 Take-off:43.5 Det Type:SDD Apollo XV Res:137 Amp.T:12.80

FS : 2727 Lsec : 74

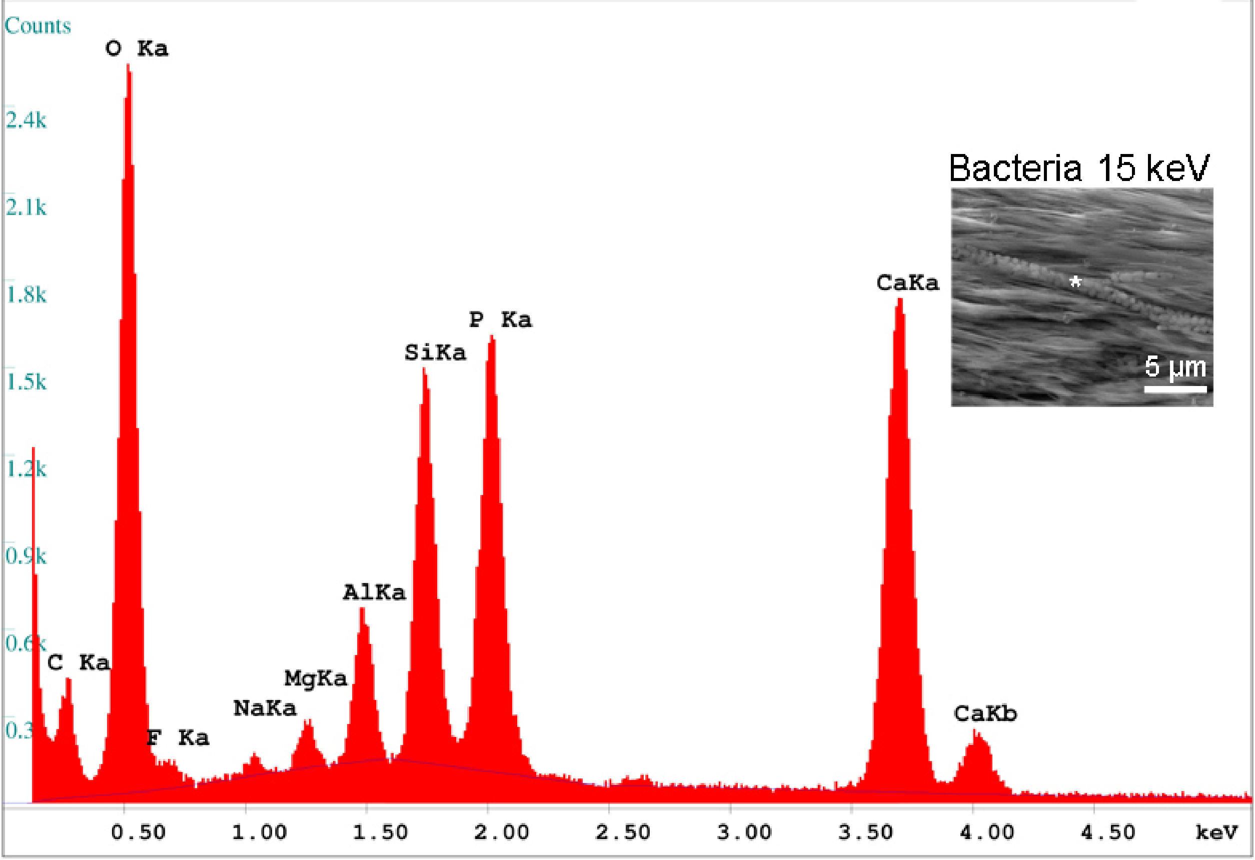

Element	Wt %	At %	Element	Net Inte.	Bkgd Inte.	Inte. Error	P/B
C K	11.16	18.96	C K	34.18	1.96	2.09	17.40
O K	39.00	49.75	O K	219.78	3.73	0.80	58.98
F K	1.85	1.99	F K	9.66	5.33	5.41	1.81
NaK	0.45	0.40	NaK	5.69	11.61	10.96	0.49
MgK	0.93	0.78	MgK	14.70	14.72	5.24	1.00
AlK	3.21	2.43	AlK	53.93	19.43	2.07	2.78
SiK	8.73	6.35	SiK	141.96	18.59	1.09	7.64
P K	11.24	7.41	P K	162.72	14.42	0.99	11.28
CaK	23.41	11.92	CaK	222.73	5.95	0.80	37.46
Total	100.00	100.00					

c:\edax32\genesis\genspc.spc

Label:

kV:5.0 Tilt:0.1 Take-off:43.5 Det Type:SDD Apollo XV Res:137 Amp.T:12.80

FS : 874 Lsec : 50

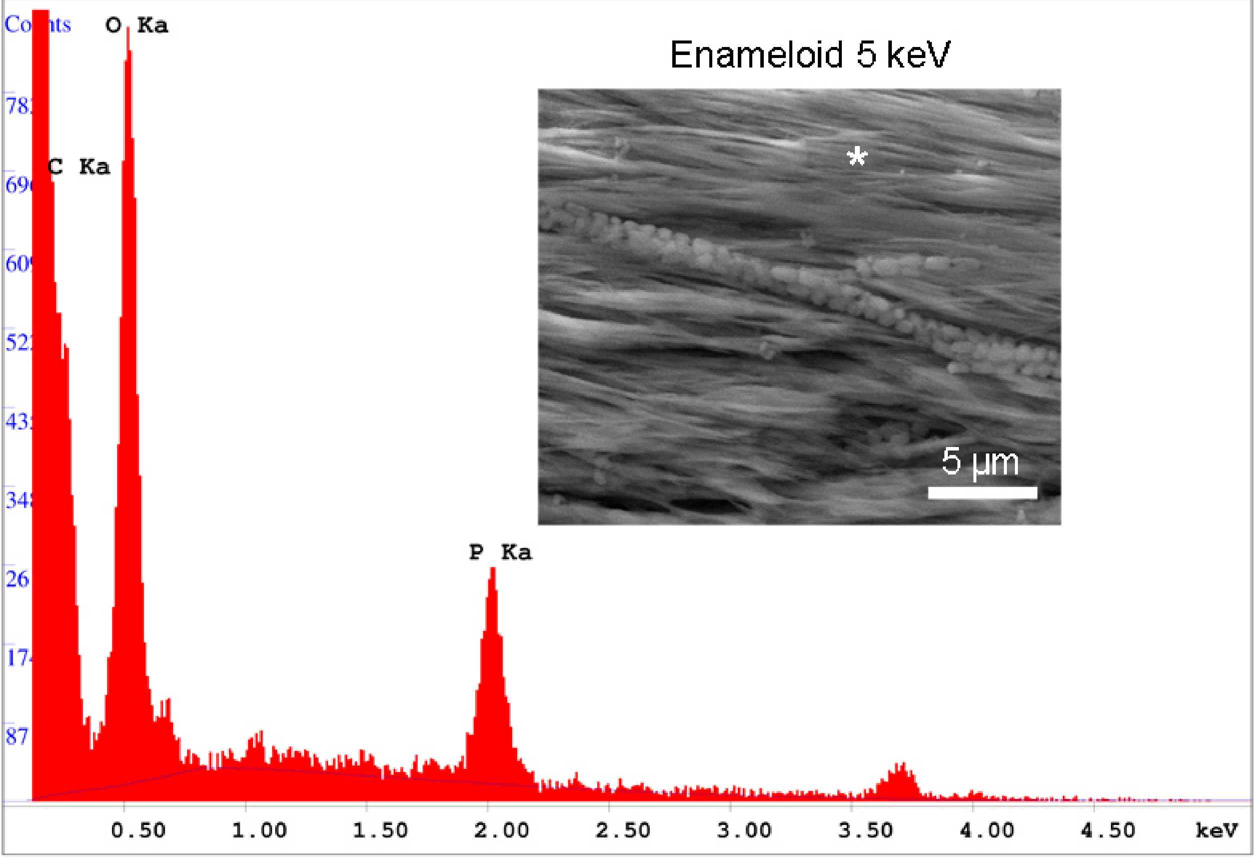

Element	Wt %	At %
C K	31.47	44.71
O K	33.99	36.26
P K	34.54	19.03
Total	100.00	100.00

Element	Net Inte.	Bkgd Inte.	Inte. Error	P/B
C K	64.61	1.52	1.80	42.59
O K	108.57	3.03	1.39	35.78
P K	37.92	3.79	2.51	10.00

c:\edax32\genesis\genspc.spc

Label:

kV:10.0 Tilt:0.1 Take-off:43.5 Det Type:SDD Apollo XV Res:137 Amp.T:12.80

FS : 1372 Lsec : 95

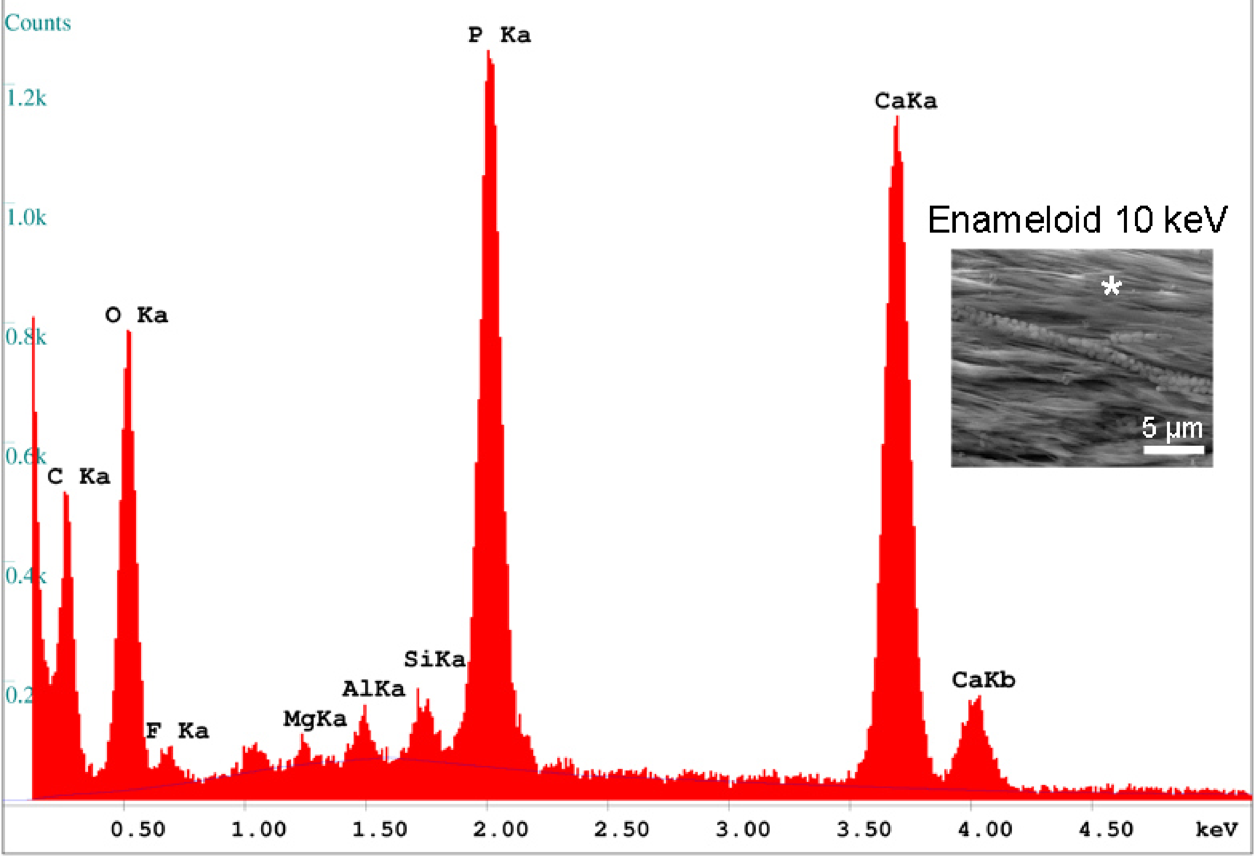

Element	Wt %	At %
C K	12.28	25.06
O K	18.46	28.26
F K	1.22	1.57
P K	19.59	15.49
CaK	48.45	29.61
Total	100.00	100.00

Element	Net Inte.	Bkgd Inte.	Inte. Error	P/B
C K	31.74	0.74	1.85	42.89
O K	51.92	1.45	1.46	35.83
F K	4.41	2.12	6.81	2.08
P K	104.79	5.94	1.05	17.64
CaK	116.85	2.64	0.97	44.31

c:\edax32\genesis\genspc.spc

Label:

kV:10.0 Tilt:0.1 Take-off:43.5 Det Type:SDD Apollo XV Res:137 Amp.T:12.80

FS : 1224 Lsec : 74

Enameloid 10 keV

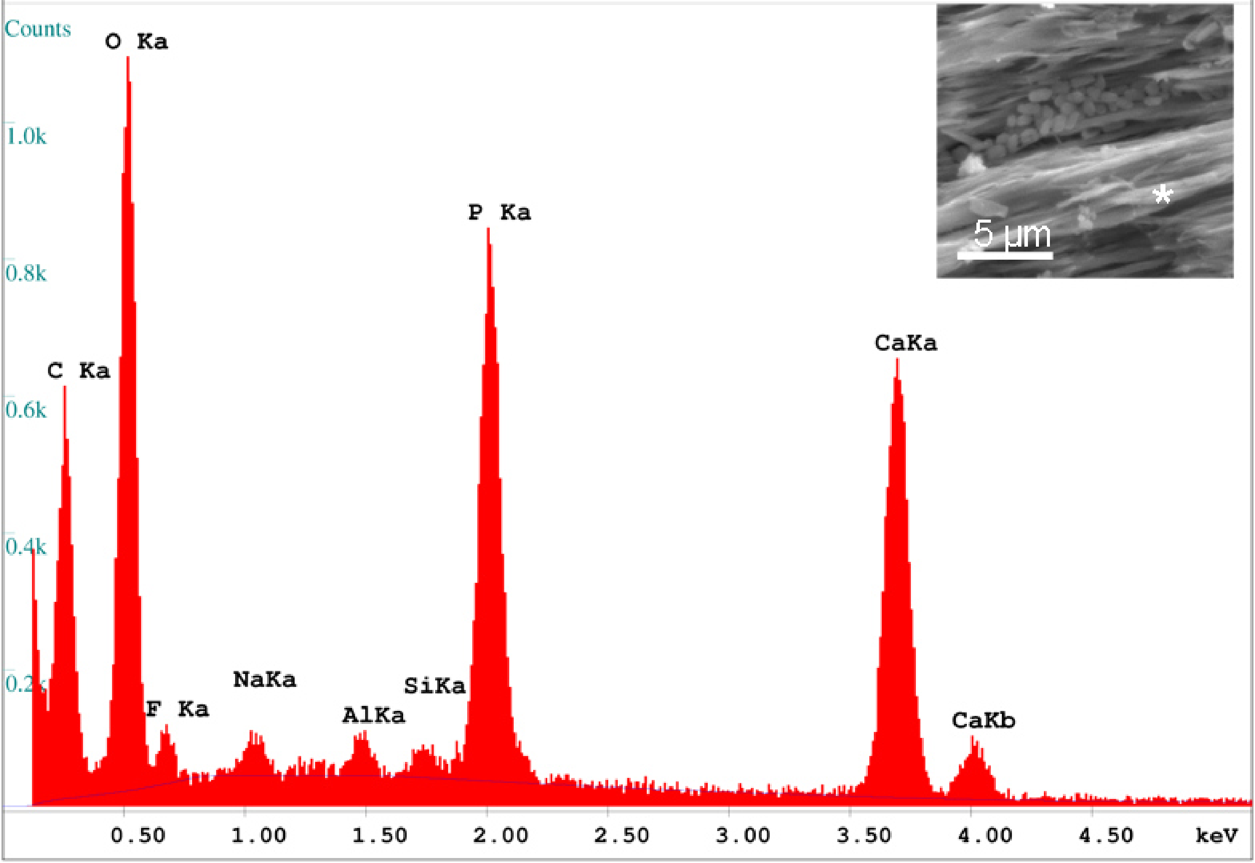

Element	Wt %	At %
C K	16.90	29.69
O K	28.78	37.96
F K	2.00	2.23
P K	16.60	11.31
CaK	35.72	18.81
Total	100.00	100.00

Element	Net Inte.	Bkgd Inte.	Inte. Error	P/B
C K	47.18	1.33	1.73	35.58
O K	94.62	2.56	1.22	36.98
F K	7.27	3.75	6.12	1.94
P K	87.75	5.16	1.31	17.01
CaK	85.22	2.14	1.28	39.76

c:\edax32\genesis\genspc.spc

Label:

kV:10.0 Tilt:0.1 Take-off:43.5 Det Type:SDD Apollo XV Res:137 Amp.T:12.80

FS : 1937 Lsec : 101

Enameloid 10 keV

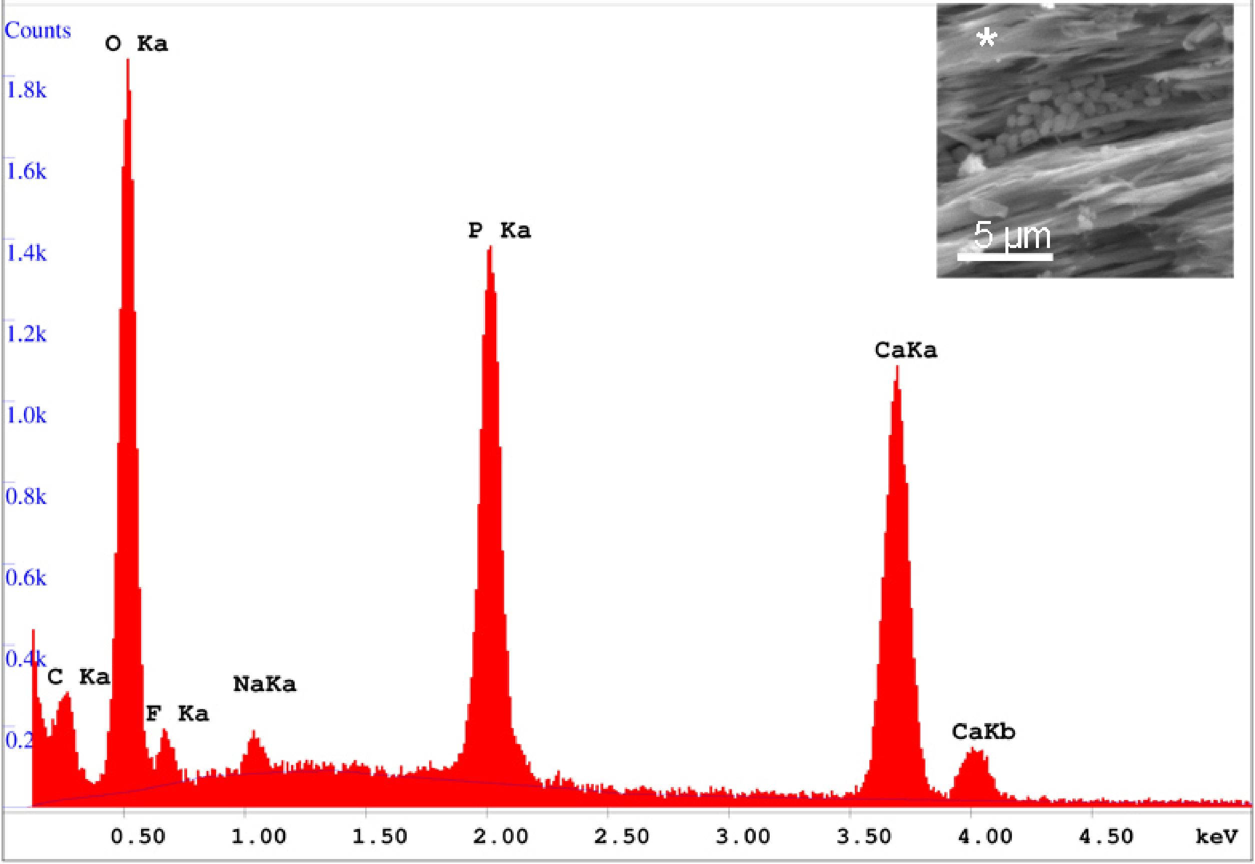

Element	Wt %	At %
C K	6.54	12.65
O K	32.66	47.43
F K	2.18	2.67
P K	19.17	14.38
CaK	39.45	22.87
Total	100.00	100.00

Element	Net Inte.	Bkgd Inte.	Inte. Error	P/B
C K	17.27	1.55	2.60	11.15
O K	112.33	2.98	0.96	37.69
F K	7.96	4.37	5.10	1.82
P K	105.28	5.80	1.02	18.14
CaK	97.99	2.22	1.03	44.13

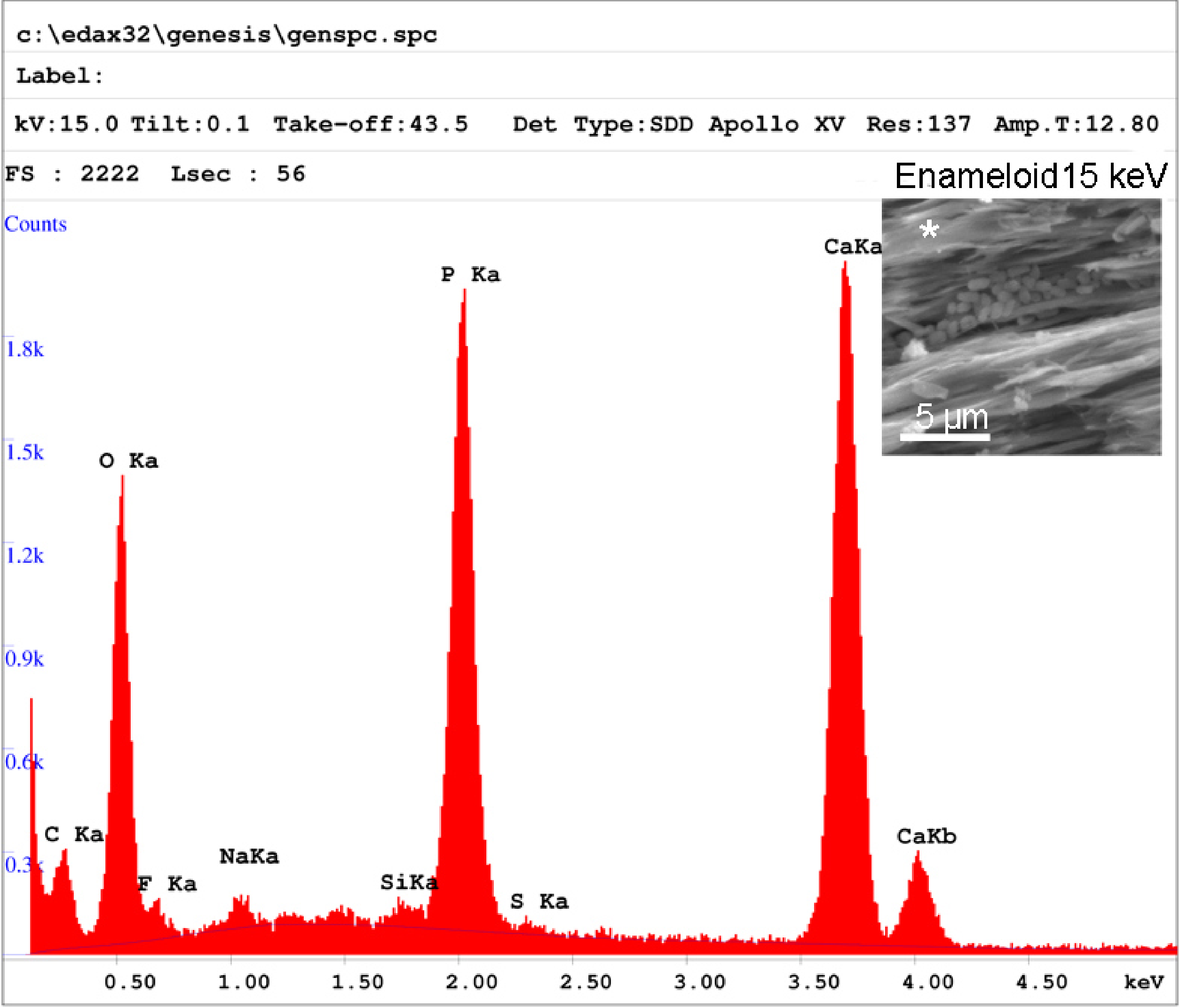

Element	Wt %	At %
C K	10.08	18.45
O K	33.74	46.36
F K	2.60	3.01
P K	17.38	12.33
CaK	36.19	19.85
Total	100.00	100.00

Element	Net Inte.	Bkgd Inte.	Inte. Error	P/B
C K	35.00	2.40	2.40	14.61
O K	157.43	4.56	1.09	34.51
F K	13.08	6.46	5.19	2.02
P K	277.69	12.78	0.84	21.73
CaK	356.25	6.30	0.72	56.54

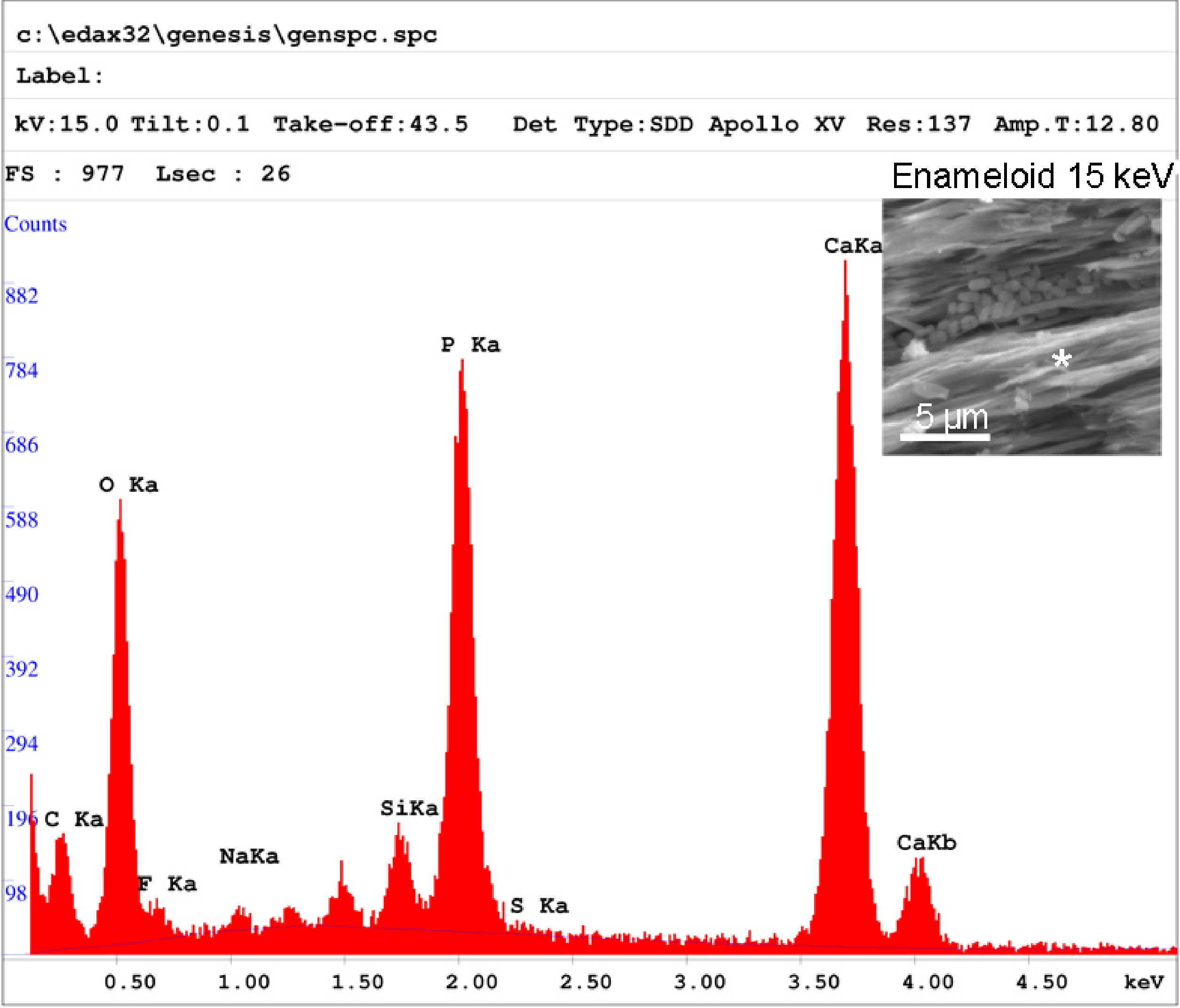

Element	Wt %	At %
C K	11.83	21.19
O K	33.86	45.52
F K	2.67	3.03
P K	16.21	11.26
CaK	35.42	19.01
Total	100.00	100.00

Element	Net Inte.	Bkgd Inte.	Inte. Error	P/B
C K	38.08	2.13	3.31	17.84
O K	141.64	4.08	1.67	34.71
F K	12.02	5.84	7.84	2.06
P K	230.75	11.20	1.33	20.61
CaK	310.96	4.64	1.11	66.98

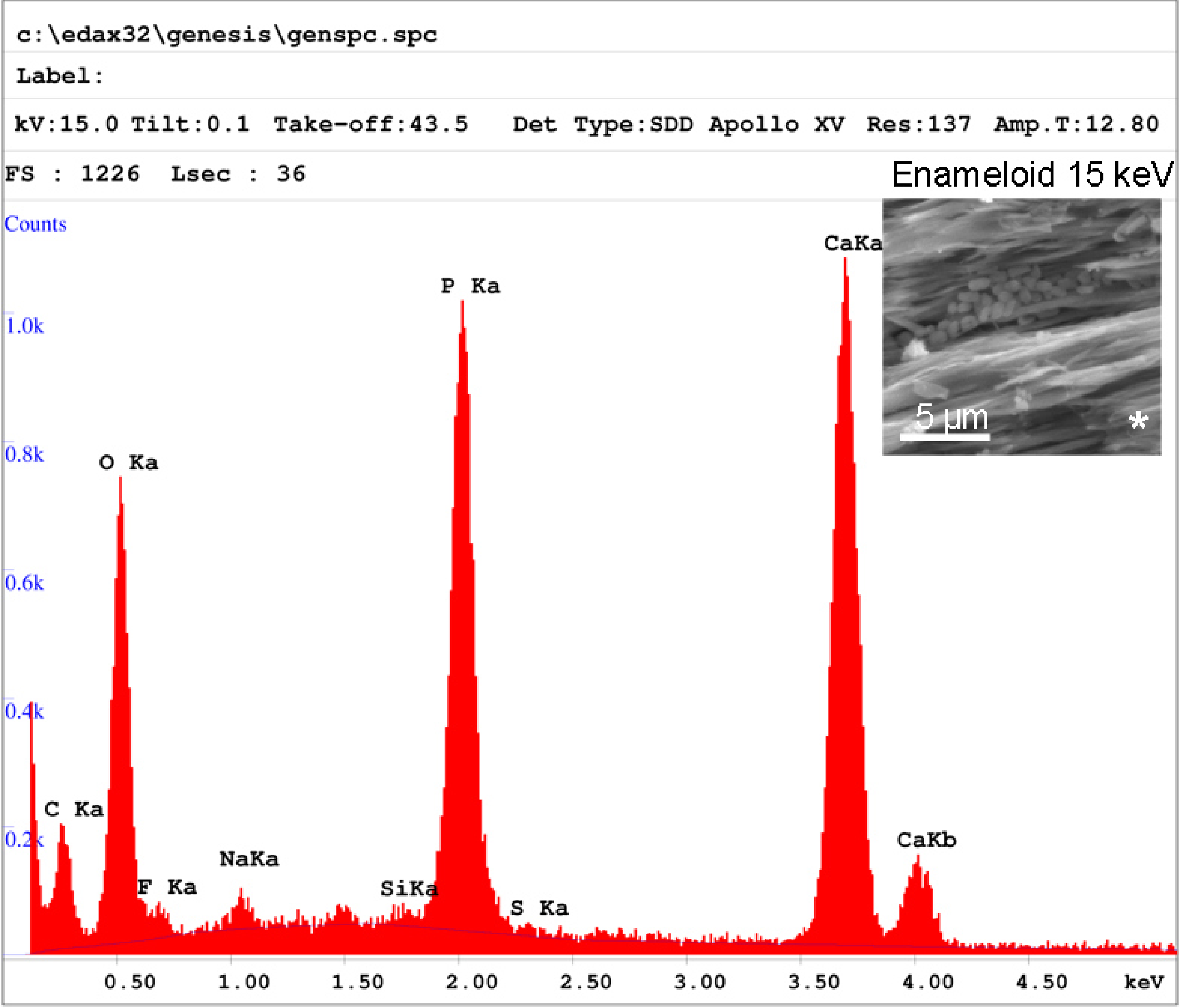

Element	Wt %	At %
C K	11.92	21.42
O K	33.35	45.00
F K	2.29	2.60
P K	17.25	12.02
CaK	35.19	18.95
Total	100.00	100.00

Element	Net Inte.	Bkgd Inte.	Inte. Error	P/B
C K	35.79	2.16	2.94	16.60
O K	133.57	4.15	1.48	32.22
F K	9.92	5.94	7.82	1.67
P K	236.30	10.83	1.13	21.81
CaK	296.63	5.03	0.98	58.97
